# An effective collaboration between parent associations and medical professionals: the Italian Pediatric Federation Audiology Network

**DOI:** 10.1186/1824-7288-40-S1-A57

**Published:** 2014-08-11

**Authors:** Jodi M Cutler, Deborah Renda, Giovanni Lenzi, Stefano Berrettini

**Affiliations:** 1 Affrontiamo la Sordita’ Insieme, Grosseto, Italy; 2 Associazione Io Sento, Palermo, Italy; 3 Pediatrician, FIMP (Italian Pediatric Federation), Grosseto, Italy; 4 University of Pisa, U.O. Otorinolaringoiatria Audiologia e Foniatria Universitaria- Pisa, Italy

## 

In the absence of a national newborn hearing screening program in Italy, parent associations have been working with the Italian Paediatric Federation (FIMP), the Society of Neonatology (SIN) and members of the Italian Society of Audiology and Phoniatrics (SIAF) to promote guidelines, best practice and training courses in early hearing detection intervention that incorporate sensitivity training for professionals working with families of deaf children. The establishment of the Italian Paediatric Federation’s Audiology Network is the result of an international collaboration between parents and medical professionals designed to promote an effective model in developing Early Hearing Detection Intervention Programs (EHDI) that recognize the role of parents as partners in the process. Among other factors, one important component frequently underestimated in most early intervention programs, both in the USA and in other countries, involves the role of parental involvement within the EHDI process. [1] From screening to identification and intervention, families must navigate through medical institutions, government and private agencies, their own family construct and community and other dynamics when raising a child who is affected by hearing loss. Family support is the “map” that keeps this process moving effectively, with sensitivity to the social and emotional needs a family will have as it adjusts to its baby’s diagnosis. The family is the social context into which children who are deaf/hard of hearing are born. The impact of a child’s hearing loss affects not only the child, but the parents, siblings, extended family and community as well.

After five years of working region by region in Italy, the network recently held its first National Pediatric Course of Audiology where the parental voice was fundamental in providing sensitivity training and in recruiting the participation of pediatricians with patients diagnosed with hearing loss.

In the absence of a Regional Protocol for Newborn Hearing Screening in Sicily, the Association Io Sento offers assistance to families of children with hearing loss. The Association collaborates with health structures and agencies to offer resources that inform, educate and support families regarding diagnosis, school services, the cochlear implant, speech habilitation by using a network created by information technology.
